# Retraction: Drivers of domestic migration in Vietnam: The characteristics of the households and their heads, environmental factors and living conditions

**DOI:** 10.1371/journal.pone.0345057

**Published:** 2026-03-17

**Authors:** The PLOS One Editors

Following publication of [1], concerns were raised that this article contains a substantial degree of overlap with a previously published article [2] by the same author with regard to reported figures, tables, methodology, references cited, datasets, and overall conclusions.

The author stated that [1] is an extension of the study reported in [2], that additional data were included in [1], and that the focus of the two articles is different. The *PLOS One* Editors consider the extent of overlap is such that this article [1] constitutes a redundant publication.

The *PLOS One* Editors retract this article [1] because, per our editorial assessment, it does not meet *PLOS One*’s publication criteria #2 that requires the reported results have not previously been published elsewhere [3].

The author did not agree with the retraction.

Tables 1-4 and Figs 3 and 5 report material previously published in [2] under a restrictive license. This material is not offered under a CC BY license and is therefore excluded from this article’s [1] license. Please provide due attribution to the original publication when referring to this content.
